# APEX1 is a novel diagnostic and prognostic biomarker for hepatocellular carcinoma

**DOI:** 10.18632/aging.102913

**Published:** 2020-03-13

**Authors:** Lei Cao, Hongwei Cheng, Qiuxia Jiang, Hui Li, Zhixian Wu

**Affiliations:** 1 Department of Hepatobiliary Disease, Dongfang Hospital, Xiamen University, Fuzhou, China; 2 The 900th Hospital of the People’s Liberation Army Joint Service Support Force, Fuzhou, China; 3 Department of Pathology, Quanzhou Women's and Children's Hospital, Quanzhou, China; 4 Institute of Chinese Medical Sciences, University of Macau, Macau SAR, China; 5 Department of Ultrasound, Quanzhou Women’s and Children’s Hospital, Quanzhou, China

**Keywords:** APEX1, overexpression, diagnosis, prognosis, alpha-fetoprotein

## Abstract

In this study, we analyzed the expression and clinical significance of apyrimidinic endodeoxyribonuclease 1 (APEX1) in hepatocellular carcinoma (HCC). The APEX1 mRNA and protein levels were significantly higher in HCC than adjacent normal liver tissues in multiple datasets from the Oncomine, GEO and TCGA databases. APEX1 levels were significantly higher in early-stage HCC patients with low alpha-fetoprotein expression. The positive predictive value (PPV) for APEX1 was significantly higher than the PPV for alpha-fetoprotein (67.91% vs. 55.22%) in HCC patients. High APEX1 expression correlated with resistance to sorafenib and anti-programmed death 1 (PD-1) therapies in HCC patients, and it associated with poorer overall survival, disease-specific survival, progression-free survival, and relapse-free survival in early- and advanced-stage HCC patients. High APEX1 expression also associated with poor prognosis in non-alcoholic, vascular invasion-negative, and hepatitis virus-negative HCC patients. These data suggest that APEX1 is a better diagnostic and prognostic biomarker than alpha-fetoprotein in HCC. Gene set enrichment analysis (GSEA) showed that APEX1 expression correlated with the DNA damage repair pathway in HCC tissues. These findings demonstrate that APEX1 is a potential diagnostic and prognostic biomarker in HCC.

## INTRODUCTION

Hepatocellular Carcinoma (HCC) is one of the most common cancers worldwide, especially in East Asia and sub-Saharan Africa [1, 2]. The major risk factors for HCC are chronic hepatitis B virus (HBV) and hepatitis C virus (HCV) infections [3, 4]. The dietary aflatoxin exposure, alcoholic abuse, age-related disorders, and metabolic disorders due to obesity are also linked to increased risk for HCC [5–7]. Antiviral therapy reduces the risk of HCC caused by chronic HBV infections [8, 9], but, elderly HCC patients show poor response to interferon-based antiviral therapy [10]. Furthermore, the incidence of HCC is significantly higher in elderly patients, probably due to reduced efficiency of physiological functions and increased susceptibility to infections [10–13]. A large number of HCC patients are diagnosed in the advanced stages because the symptoms are not evident during early stages. Moreover, the survival rates of these patients are poor despite the availability of curative treatments because of increased recurrence [14]. Hence, effective early diagnostic markers and novel therapeutic targets are necessary to improve the overall survival rates and disease prognosis of HCC patients. The gold standard diagnostic marker for HCC is alpha-fetoprotein (AFP). However, its sensitivity and specificity is low and its expression can be influenced by several non-HCC related factors [15, 16]. Hence, novel sensitive biomarkers are urgently needed for early identification of HCC and improve the clinical outcomes. Previous studies have identified glypican 3 (GPC3), Golgi protein-73 (GP73), descarboxyprothrombin (DCP), glutamic—pyruvic transaminase (GPT) and gamma-glutamyl carboxylase (GGCX) as complementary biomarkers for HCC diagnosis [17–19]. Nevertheless, the early and specific diagnosis of HCC remains challenging.

Apyrimidinic endodeoxyribonuclease 1 (APEX1) is involved in the DNA damage response and is expressed in a wide array of human tissues [20]. Aberrant expression of APEX1 can disrupt several physiological processes, including cellular redox homeostasis, smooth muscle cell migration, cell cycle, apoptosis, and mRNA stability [21]. The N-terminal APEX1 domain is responsible for the redox regulation of transcription factors, such as p53 [22], c-Fos and c-Jun [23], and Myb [24]. The C-terminal domain is critical for the DNA repair activity of APEX1 [25]. In some cancers, APEX1 is aberrantly expressed and activates the Notch signaling pathway [20]. The expression of APEX1 positively correlates with the prognosis of osteosarcoma patients [26]. Furthermore, high expression of APEX1 is associated with chemotherapeutic resistance in biliary cancer, and hence considered as a potential therapeutic target to sensitize tumors to chemotherapy [27].

In this study, we investigated the expression and clinical significance of APEX1 in HCC.

## RESULTS

### APEX1 is overexpressed in hepatocellular carcinoma

**Figure 1:**
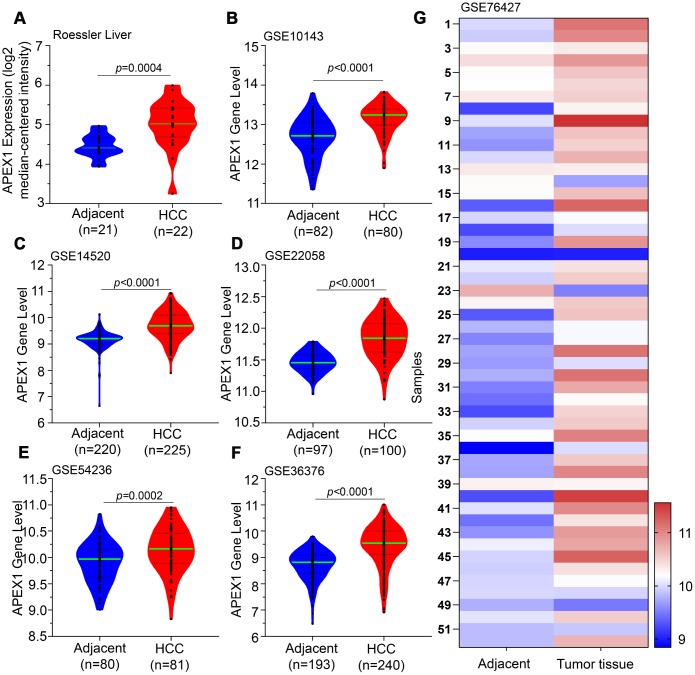
**APEX1 transcript levels in HCC and adjacent normal liver tissues.** The APEX1 transcript levels in HCC and adjacent normal liver tissues from the (**A**) Roessler Liver dataset (22 HCC and 21 normal liver samples) and (**B**) GSE10143; (**C**) GSE14520; (**D**) GSE22058; (**E**) GSE54236; and (**F**) GSE36376 datasets are shown. (**G**) The heatmap shows APEX1 mRNA expression in 52 paired HCC and corresponding adjacent normal liver tissues.

We observed significantly higher expression of APEX1 mRNA in the HCC tissues compared to the normal liver tissues in the Roessler Liver dataset from the Oncomine database (Figure 1A). Moreover, APEX1 mRNA levels were significantly higher in the HCC tissues compared to the adjacent normal liver tissues in multiple GEO datasets (Figure 1B–1G). These results demonstrate that APEX1 is overexpressed in HCC.

### The expression of APEX1 was positively correlated with the development of hepatocellular carcinoma

**Figure 2:**
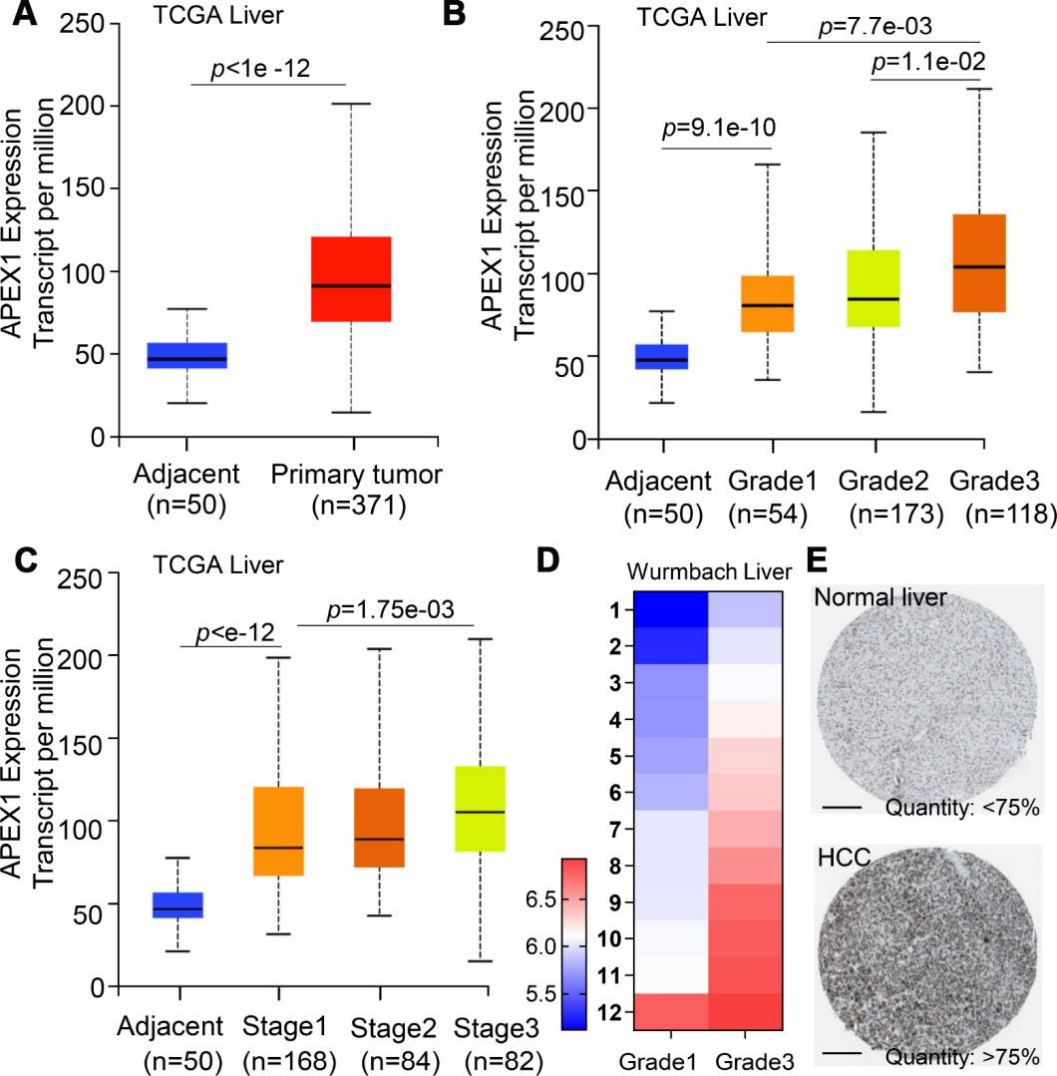
**APEX1 mRNA and protein expression correlates with HCC tumor grades.** (**A**) APEX1 mRNA levels in 371 HCC and 50 normal liver tissues from the TCGA database are shown. (**B**) The histogram plot shows APEX1 mRNA expression in grades 1-4 HCC patients. As shown, APEX1 mRNA expression is incrementally upregulated with increasing tumor grades. (**C**) The histogram plot shows APEX1 mRNA expression levels in stages 1-4 HCC patients. As shown, APEX1 mRNA levels show incremental upregulation with increasing tumor stages. (**D**) The heat map shows APEX1 mRNA levels in grade 1 and 3 HCC patients in the Wurmbach Liver dataset from the Oncomine database. (**E**) Representative images show APEX1 protein expression in HCC and adjacent normal liver tissues that were obtained from The Human Protein Atlas database. The APEX protein expression was analyzed by immunohistochemistry. The scale bar is 200μm.

APEX1 was overexpressed in the HCC tissues compared to normal liver tissues in The Cancer Genome Atlas (TCGA) database (Figure 2A). Next, we analyzed APEX1 expression in HCC specimens from different tumor stages in the TCGA database. We observed incremental increase in the APEX1 mRNA levels with higher tumor grades and stages (Figure 2B, 2C). The expression of APEX1 was significantly higher even in the early-stages of HCC compared with the normal liver tissues (Figure 2B, 2C). APEX1 mRNA levels were significantly higher in grade 3 HCC samples than in the grade 1 HCC tissues from the Wurmbach liver dataset from the Oncomine database (Figure 2D and Supplementary Figure 1). Furthermore, analysis of the Human Protein Atlas database showed that the APEX1 protein levels in the HCC tissues were 75% higher quantity than in the normal liver tissue samples (Figure 2E).

### APEX1 is a diagnostic biomarker for HCC

**Figure 3:**
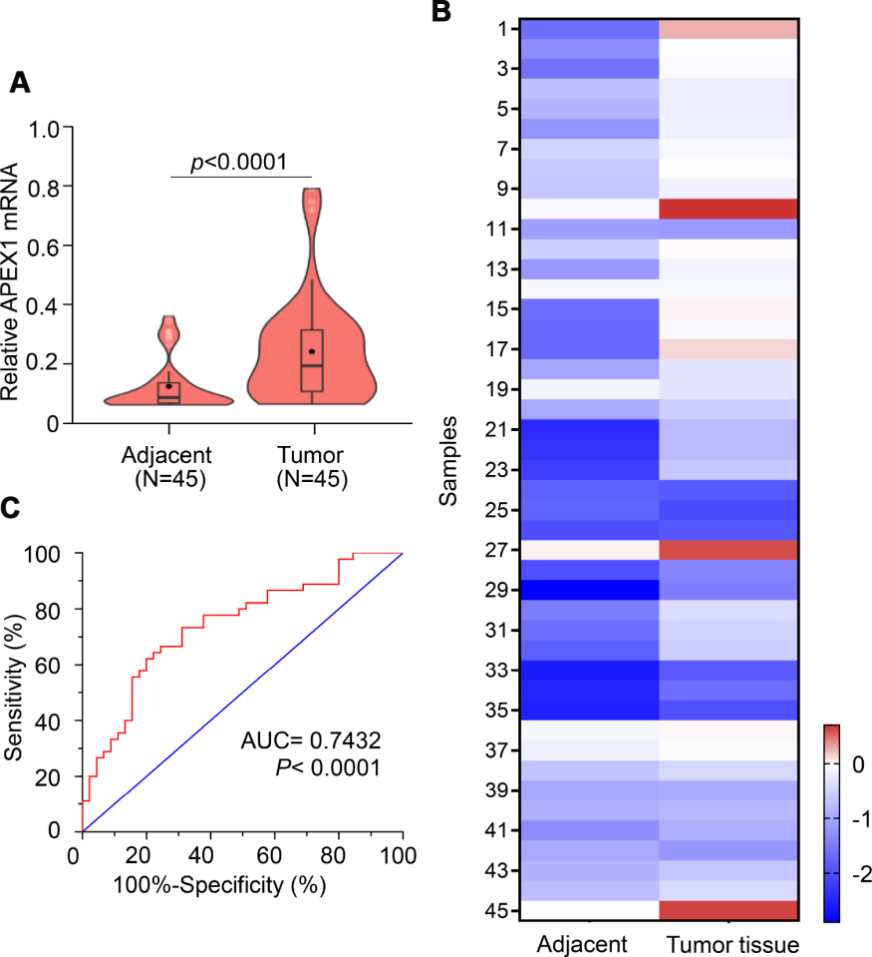
**APEX1 is a potential diagnostic biomarker for HCC.** (**A**) The violin plot, (**B**) heatmap and (**C**) ROC curve analysis shows APEX1 mRNA levels in tumor and adjacent normal liver tissues isolated from 45 HCC patients.

QRT-PCR analysis of 45 paired HCC patient samples showed that APEX1 mRNA levels were significantly higher in tumor tissues compared to paired adjacent normal liver tissues (Figure 3A). Among these, APEX1 expression in HCC tissues was five-fold higher in 19 cases and 1.5-fold higher in 15 cases compared with their corresponding adjacent normal liver tissues. The mean APEX1 expression in the 45 HCC tissue samples was 75% higher than in the corresponding adjacent normal liver tissue samples (Figure 3B). These data demonstrate that APEX1 is highly expressed in HCC tissues. Based on these results, we hypothesized that APEX1 is a diagnostic biomarker for HCC. ROC curve analysis demonstrated that APEX1 was diagnostically significant with an AUC value of 0.7432 (Figure 3C). This strongly suggests that APEX1 is a potential diagnostic biomarker for HCC.

### APEX1 shows higher positive predictive value than AFP for HCC diagnosis

**Figure 4:**
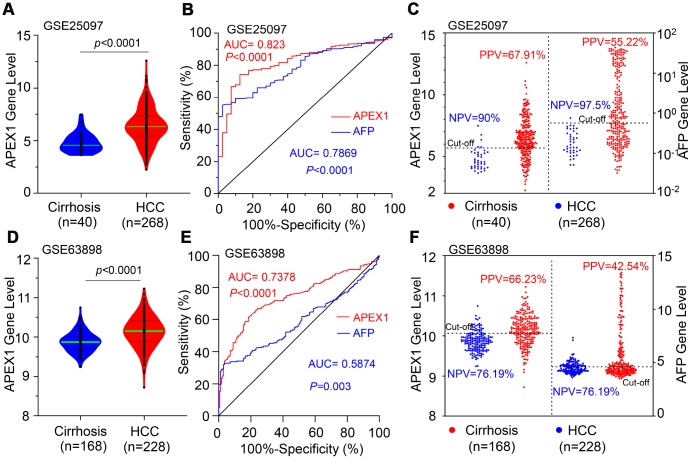
**APEX1 shows a higher positive predictive value than AFP in HCC patients.** (**A**) The histogram plot shows APEX1 mRNA levels in patients with liver cirrhosis (n=40) and HCC (n=268) from the GSE25097 dataset. (**B**) ROC curve analysis shows the diagnostic value of APEX1 and AFP in liver cirrhosis and HCC patients from the GSE25097 dataset. (**C**) The optimal cut-off value for APEX1 and AFP in liver cirrhosis and HCC patients was determined according to the Youden and Product indices. Also shown are the positive predictive value (PPV) and negative predictive value (NPV) for APEX1 and AFP. (**D**) The histogram plot shows relative APEX1 mRNA levels in patients with liver cirrhosis (n=168) and HCC (n=228) from the GSE63898 dataset. (**E**) ROC curve analysis shows the diagnostic values of APEX1 and AFP in the liver cirrhosis and HCC patients from the GSE63898 dataset. (**F**) The optimal cut-off values for APEX1 and AFP in the liver cirrhosis and HCC patients was determined according to the Youden and Product indices. Also shown are the PPV and NPV for APEX1 and AFP.

Next, we analyzed the GSE25097 and GSE63898 datasets using ROC analysis to distinguish HCC from liver cirrhosis patients. Both APEX1 and AFP expression was significantly higher in HCC tissues compared to liver cirrhosis samples (Figure 4A and Supplementary Figure 2). The AUC value of 0.823 for APEX1 (95% CI: 0.765 to 0.881; p<0.001) was significantly higher than the AUC value of 0.7869 for AFP in HCC tumor samples (95% CI: 0.7295 to 0.8443, p<0.0001) as shown in Figure 4B. Then, we determined the best cut-off values for APEX1 and AFP based on the specificity and sensitivity of the ROC curve. The negative predictive values (NPV) for APEX1 (90%) and AFP (97.5%) were statistically similar, but, the positive predictive value (PPV) of APEX1 (67.91%) was significantly higher than the PPV for AFP (55.22%) as shown in Figure 4B, 4C. This demonstrates that APEX1 was a more sensitive diagnostic marker than AFP in HCC patients. Next, we performed ROC analysis of HCC and liver cirrhosis samples using the GSE63898 dataset. In the GSE63898 dataset, the APEX1 mRNA levels of the HCC tissues were significantly higher than the liver cirrhosis samples (Figure 4D). Moreover, the AUC value for AFP was 0.5874 (95%CI, 0.53 to 0.64; p=0.003), which was significantly lower than the AUC value of 0.7378 for APEX1 (95%CI, 0.69 to 0.79, p<0.0001) for the HCC samples (Figure 4E). Moreover, the NPV for AFP was similar to that of APEX1, but, the PPV for APEX1 (66.23%) was significantly higher than the PPV for AFP (42.54%) (Figure 4F). These results demonstrate that APEX1 was a more sensitive diagnostic marker than AFP to distinguish HCC patients from those with liver cirrhosis.

### APEX1 shows better diagnostic value in HCC patients with low AFP expression

**Figure 5:**
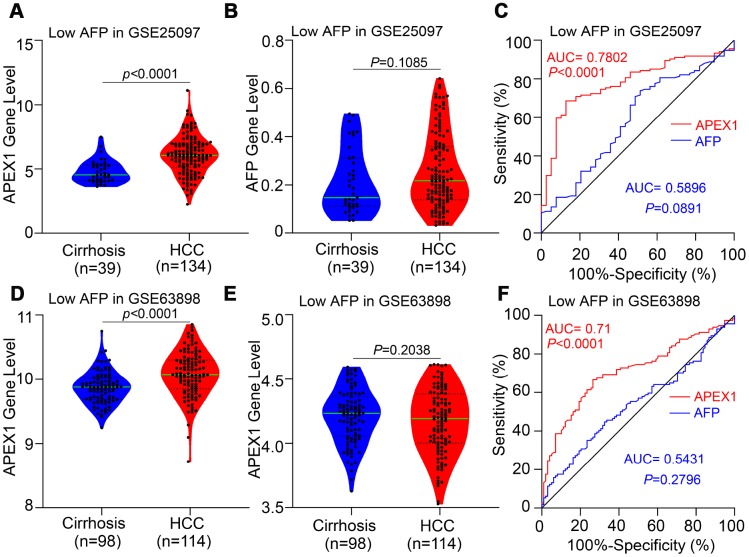
**APEX1 expression is significantly higher in the HCC patients with low AFP expression and is a better diagnostic biomarker.** (**A**, **B**) APEX1 and AFP mRNA expression in the liver cirrhosis (n=40) and HCC patients with low AFP expression (n=134) from the GSE25097 dataset is shown. The 134 HCC patients with low AFP expression were selected based on the median expression of AFP in 268 HCC patients from the GSE25097 dataset. (**C**) ROC curve analysis shows the diagnostic values of APEX1 and AFP in 134 HCC patients with low AFP expression from the GSE25097 dataset. (**D**, **E**) APEX1 and AFP mRNA expression in the liver cirrhosis (n=168) and HCC patients with low AFP expression (n=114) from the GSE63898 dataset is shown. The 114 HCC patients with low AFP expression were selected based on the median expression of AFP in 228 HCC patients in the GSE63898 dataset. (**F**) ROC curve analysis shows the diagnostic value of APEX1 and AFP in 114 HCC patients with low AFP expression from the GSE63898 dataset.

HCC patients with low AFP expression cannot be distinguished from those with liver cirrhosis because of low sensitivity of AFP. Therefore, we evaluated the diagnostic value of APEX1 in HCC patients with low AFP expression using the GSE25097 and GSE63898 datasets. The APEX1 levels were significantly higher in low-AFP expressing HCC patients compared to patients with liver cirrhosis (Figure 5A and 5D). As expected, the AFP levels of liver cirrhosis and HCC patients were similar in these two datasets (Figure 5B and 5E), and the AUC value of AFP in the GSE25097 and GSE63898 datasets were not statistically significant (Figure 5C and 5F). The ROC curve analysis showed that the AUC values for APEX1 were statistically significant in both datasets (GSE25097: AUC, 0.7802, p<0.0001; GSE63898: AUC, 0.71, p<0.0001; Figure 5C and 5F). These data demonstrate that APEX1 can be used to accurately diagnose low AFP-expressing HCC patients that cannot be distinguished from those with liver cirrhosis using AFP.

### APEX1 is a better diagnostic biomarker in early stage HCC patients

**Figure 6:**
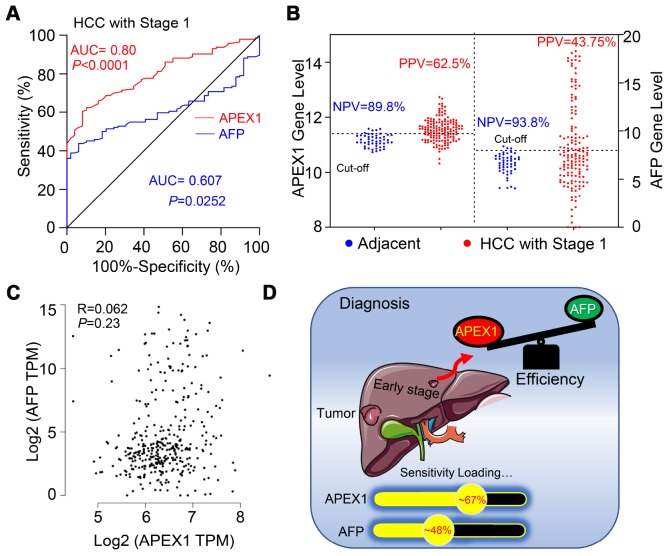
**APEX1 is a better diagnostic biomarker than AFP in early stage HCC patients.** (**A**) The diagnostic values of APEX1 and AFP in stage 1 HCC patients based on ROC curve analysis is shown. (**B**) The best cut-off values for APEX1 and AFP were calculated using the Youden index in combination with the Product index. (**C**) The analysis of Pearson correlation co-efficients for APEX1 and AFP expression in the HCC and adjacent normal liver tissues is shown. (**D**) The graphical representation shows the mean diagnostic sensitivity of APEX1 and AFP in HCC patients from the GSE25097 and GSE63898 datasets. APEX1 shows significantly higher diagnostic value compared to AFP.

Next, we analyzed the diagnostic value of APEX1 in stage 1 HCC patients using ROC analysis. As shown in Figure 6A, the diagnostic value of APEX1 (AUC: 0.80, 95%CI: 0.738-0.862, p<0.0001) was significantly higher than that of AFP (AUC: 0.607, 95%CI: 0.531-0.683, p=0.0252). Moreover, the positive predictive value (PPV) of APEX1 was significantly higher than that of AFP (62.5% vs. 43.75%; Figure 6B). These results suggest that APEX1 is a more sensitive biomarker than AFP in the early diagnosis of HCC. Pearson correlation analysis showed no association between APEX1 and AFP in HCC patients (Pearson R=0.062, p=0.23; Figure 6C). Moreover, APEX1 showed no correlation with other potential biomarkers such as DCP, GP73, GPC3, and GPT (Supplementary Figure 3). These data indicate that APEX1 is an independent diagnostic biomarker in HCC.

### High APEX1 expression correlates with drug resistance in HCC patients

**Figure 7:**
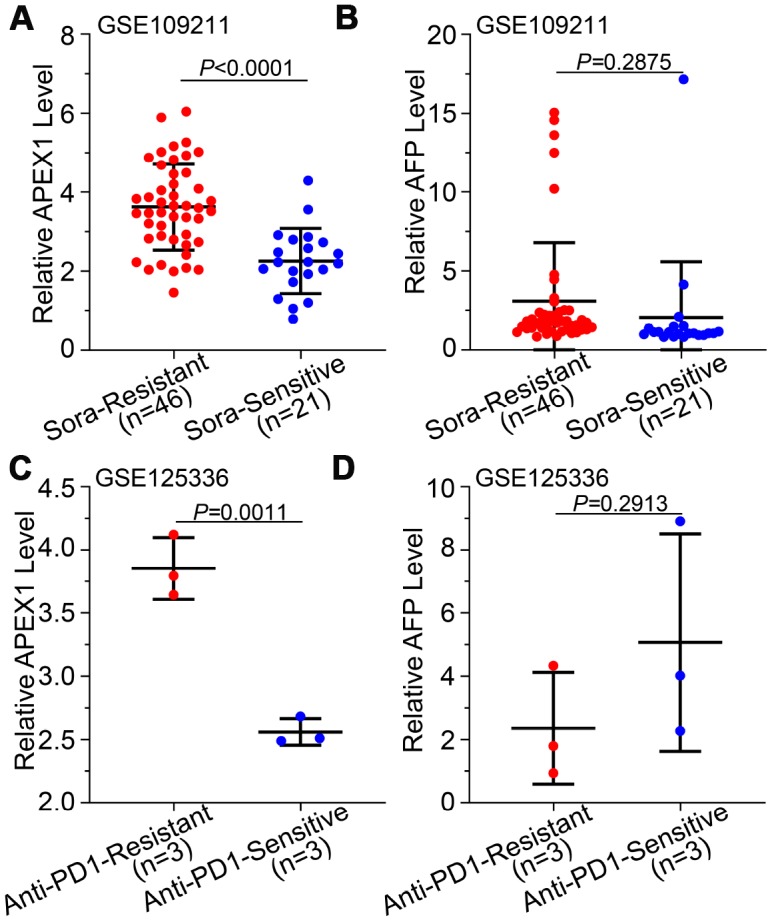
**High APEX1 expression correlates with resistance against sorafenib and anti-PD1 immunotherapy in HCC patients.** (**A**, **B**) APEX1 and AFP mRNA expression in 46 sorafenib-resistant and 21 sorafenib-sensitive HCC patients from the GSE109211 dataset is shown. (**C**, D) APEX1 and AFP expression in three anti-PD1 immunotherapy-resistant and three anti-PD1 immunotherapy-sensitive HCC patients belonging to the GSE125336 dataset is shown.

Drug resistance is an important factor that determines poor prognosis of HCC patients. We analyzed the correlation between APEX1expression and drug resistance in HCC patients. Sorafenib is the gold standard main chemotherapeutic drug for treating advanced stage HCC patients, but its efficacy is affected by drug resistance [28]. We evaluated the correlation between APEX1 and sorafenib-resistance in HCC patients from the GSE109211 database. APEX1 expression was significantly higher in the sorafenib-resistant HCC patients than in the sorafenib-sensitive HCC patients (Figure 7A). However, the AFP levels of both sorafenib-sensitive and –resistant groups were similar (Figure 7B). Next, we analyzed the correlation between APEX1 expression and tolerance against PD-1 immunotherapy in HCC patients. APEX1 expression was significantly higher in HCC patients showing resistance against PD-1 immunotherapy compared to those sensitive to PD-1 immunotherapy (Figure 7C). However, AFP expression was similar in both PD-1 immunotherapy-resistant and –sensitive groups (Figure 7D). These results demonstrate that APEX1 expression correlates with drug resistance in HCC patients.

### APEX1 expression correlates with prognosis of HCC patients

**Figure 8:**
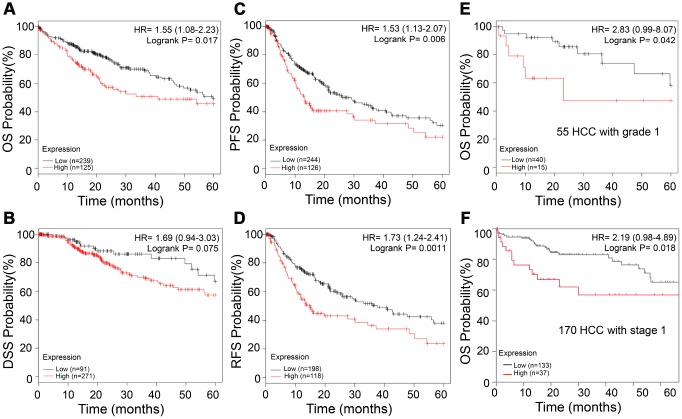
**High APEX1 expression predicts poor prognosis in HCC patients, including those with grade 1 tumors.** (**A–D**) Kaplan-Meier survival curve analysis shows OS, DSS, PFS and RFS rates of high and low APEX1 expressing HCC patients, respectively. (**E**) Kaplan-Meier survival curve analysis of 5-year OS probability of 55 tumor grade 1 HCC patients is shown. (**F**) Kaplan-Meier survival curve analysis of the 5-year OS rate of HCC patients with stage 1 cancer is shown. The hazard ratio (HR) was calculated based on the Cox Proportional-Hazards model.

We then evaluated the prognostic significance of APEX1 in HCC patients. As shown in Figure 8A–8D, HCC patients with high APEX1 expression were associated with worse overall survival (OS, log-rank p=0.017), disease-specific survival (DSS, log-rank p=0.075), progression-free survival (PFS, log-rank p=0.006), and relapse-free survival (RFS, log-rank p=0.0011). Next, we analyzed the prognostic significance of APEX1 in early stage HCC patients. As shown in Figure 8E–8F, grade 1 or stage 1 HCC patients in the high APEX1 expression group were associated with worse OS (grade 1 HCC: HR=2.83, log-rank p=0.042; stage 1 HCC: HR=2.19; log-rank p=0.018) compared to those in the low APEX1 expression group. These results demonstrate that APEX1 is a potential prognostic biomarker in HCC patients, including those with early stage HCC.

### APEX1 expression correlates with prognosis in non-alcoholic, vascular invasion-negative and hepatitis virus infection-negative HCC patients

**Figure 9:**
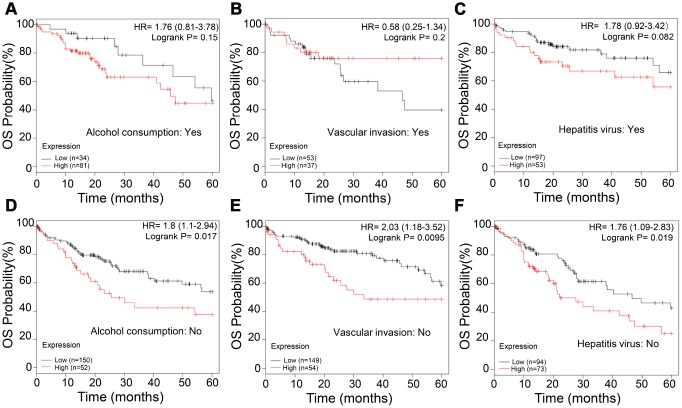
**APEX1 is a prognostic biomarker in non-alcoholic, vascular invasion-negative and hepatitis virus infection-negative HCC patients.** (**A**, **B**) Kaplan-Meier survival curve analysis shows the OS rates of high- and low-APEX1 expressing alcoholic and non-alcoholic HCC patients. (**C**, **D**) Kaplan-Meier survival curve analysis shows the OS rates of high- and low-APEX1 expressing vascular invasion-positive and vascular invasion-negative HCC patients. (**E**, **F**) Kaplan-Meier survival curve analysis shows the OS rates of high- and low-APEX1 expressing hepatitis virus infection-positive and hepatitis virus infection-negative HCC patients.

Next, we analyzed the correlation between APEX1 expression and various clinical characteristics of HCC patients, including alcohol consumption, vascular invasion, and hepatitis virus infection. The OS rates were similar in high- and low-APEX1 expressing HCC patients that belong to alcohol consuming, vascular invasion-positive, and hepatitis virus infection-positive groups (Figure 9A–9C). However, high APEX1 expressing HCC patients belonging to non-alcohol consuming, vascular invasion-negative and hepatitis virus infection-negative groups showed significantly worse OS rates than those with low APEX1 expression (Figure 9D–9F; Supplementary Figure 4, log-rank p=0.026). These data suggests that APEX1 is a valuable prognostic biomarker for HCC patients belonging to non-alcohol consuming, vascular invasion-negative and hepatitis virus infection-negative groups.

### High APEX1 expression correlates with DNA damage repair signaling in HCC

**Figure 10:**
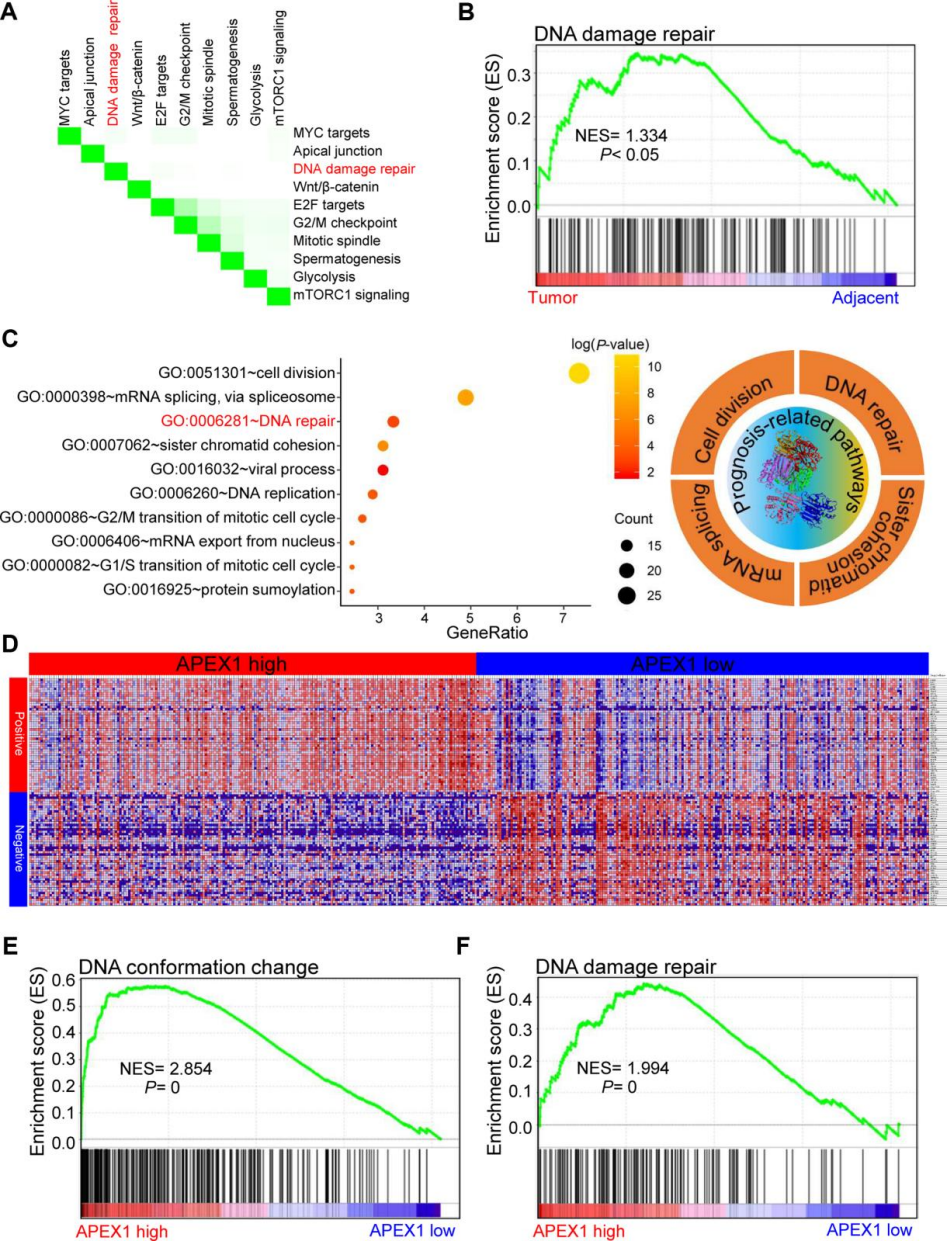
**High APEX1 expression positively correlates with DNA damage repair in HCC patients.** (**A**) The top ten signaling pathways in HCC tissues based on gene set enrichment analysis (GSEA) are shown. (**B**) Enrichment plot shows the status of DNA damage repair pathways in HCC and adjacent normal liver tissue samples. (**C**) The most significant survival-associated genes in the HCC tissues according to analysis using the GEPIA portal and the most enriched genes in HCC tissues based on the gene ontology (GO) analysis are shown. The right panel shows the top four prognosis-related pathways that are enriched in the HCC tissues. (**D**) The median mRNA levels of genes that co-express with APEX1 in HCC tissues are shown. (**E**, **F**) The enrichment plot shows GSEA analysis of genes involved in DNA conformation change and DNA damage repair in the HCC tissues.

Previous studies have shown that APEX1 is actively involved in the DNA damage response in lung [29] and prostate [30] cancers. Moreover, DNA damage repair pathways play an important role in the growth and progression of several cancers [31]. Gene set enrichment analysis (GSEA) analysis of paired HCC and adjacent normal liver samples showed that the top ten differentially regulated pathways were MYC, DNA damage repair, Wnt/β-catenin, E2F, G2/M checkpoint, mitotic spindle, glycolysis, and mTORC1 signaling (Figure 10A). The normalized enrichment score for the DNA damage repair pathway was 1.334 (Figure 10B), which suggests that DNA damage repair positively correlates with HCC growth and progression. We then analyzed the signaling pathways associated with prognosis or survival of HCC patients. The list of significant survival-associated genes in HCC tissues are shown in Supplementary Table 1 with GEPIA portal. Gene ontology (GO) analysis demonstrates that the DNA repair pathway significantly correlates with the prognosis of HCC patients (Figure 10C). The genes that co-express with APEX1 in HCC tissues are shown in Figure 10D. High APEX1 expression in HCC tissues positively correlates with pathways related to DNA conformational change and DNA damage repair (Figure 10E, 10F). These results show that APEX1 expression positively correlates with the DNA damage repair signaling pathway in HCC patients.

## DISCUSSION

HCC ranks 14^th^ among the cancers with newly diagnosed cases in a year and 5^th^ among the cancer- related mortality according to the 2019 cancer statistics reported in the United States [32]. Sorafenib is the only FDA-approved chemotherapeutic drug for advanced stage HCC [28]. There is an urgent need for novel therapeutic options for HCC to overcome the poor survival outcomes because of fewer effective drugs, chemotherapeutic resistance, and radiation toxicity. Furthermore, most patients are diagnosed in the advanced stages and are not amenable for surgery. Hence, there is an urgent need for effective early diagnostic markers to improve the prognosis of HCC patients. While aberrant APEX1 expression has been reported in some tumors including prostate cancer, colon cancer and so on [20, 30], its role in HCC is relatively unknown. Therefore, we aimed to study the expression and the clinical significance of APEX1 in HCC. Our study shows that APEX1 is overexpressed in early and advanced stage HCC tumor tissues (Figures 1–3). Besides, APEX1 expression positively correlates with HCC initiation and progression (Figure 2A–2D). These results suggest that APEX1 is a potential biomarker for HCC. ROC curve analysis demonstrates that the positive predictive value of APEX1 was better than that of AFP, the current gold standard biomarker for HCC diagnosis (Figure 4). We also demonstrate that APEX1 is highly expressed in early stage HCC patients (Figure 2B, 2C) and is a more sensitive diagnostic biomarker in HCC patients with low AFP expression (Figure 5). The diagnostic sensitivity of APEX1 is significantly higher than AFP (62.5% vs. 43.75%; Figure 6B). These results demonstrate that APEX1 is a very effective diagnostic biomarker for HCC, especially for early stage HCC patients with low AFP expression. Moreover, correlation analysis shows that APEX1expression is an independent diagnostic factor for HCC (Figure 6C and Supplementary Figure 3).

The efficacy of AFP in determining the prognosis of HCC patients is poor [33]. Our data suggests that APEX1 expression correlates with response to sorafenib and anti-PD-1 therapies in HCC patients. APEX1 expression is significantly higher in patients that develop resistance to these two therapies, whereas AFP levels are similar in both drug-resistant and -sensitive HCC patients (Figure 7). This indicates that APEX1 expression can be used to evaluate the response to sorafenib and PD-1 immunotherapy. However, it should be noted that we used biopsy samples for our study. Other diagnostic methods like ultrasonography, Computed tomography (CT) and magnetic resonance imaging (MRI) are more convenient than biopsy for liver disease [34]. A recent report shows that serum APEX1 can be used as a biomarker for cholangiocarcinomas [35]. It is plausible that serum APEX1 levels may be elevated in HCC patients and may be a more sensitive diagnostic biomarker. However, this needs to be investigated in future studies.

We also demonstrate that APEX1 is a potential prognostic biomarker for HCC patients, even for those with early stage HCC (Figure 8). Moreover, high expression of APEX1 is prognostically significant for HCC patients that do not consume alcohol and belong to vascular invasion-negative and hepatitis virus infection-negative groups (Figure 9). Aberrant regulation of the DNA damage repair pathway plays an important role in oncogenesis [31]. Our study demonstrates that DNA damage repair pathway is significantly enriched in HCC tissues (Figure 10A–10C). APEX1 is a known regulator of DNA damage repair [30]. Our study shows a positive correlation between APEX1 and DNA damage repair signaling pathway (Figure 10D–10F). Hence, we postulate that APEX1 plays a role in DNA damage repair in the HCC tissues.

In conclusion, our study demonstrates that APEX1 is a novel diagnostic biomarker for HCC, particularly for early stage patients and patients with low AFP expression. Our study also demonstrates that APEX1 is a potential prognostic biomarker for HCC patients, including those that belong to non-alcohol consuming, vascular invasion-negative and hepatitis virus infection-negative groups. APEX1 expression correlates with the DNA damage repair signaling pathway in HCC tumor tissues. Overall, our study demonstrates that APEX1 is a potential diagnostic and prognostic biomarker for HCC.

## MATERIALS AND METHODS

### Gene expression analysis

We analyzed APEX1 mRNA levels of the Roessler Liver 2 dataset in the Oncomine database by applying a threshold as a p-value < 1E-4, fold change ≥ 2.0, and the gene ranking in the top 10. The Roessler Liver statistics were based on the Affymetrix Human Genome HT U133A 2.0 microarray platform [36]. We analyzed APEX1 expression in HCC patients with different tumor grades using the Wurmbach Liver dataset that was based on the Affymetrix Human Genome U133 plus 2.0 microarray platform [37]. We also analyzed APEX1 and AFP mRNA expression in the HCC and control samples using the GSE10143 [38], GSE14520 [36], GSE22058 [39], GSE54236 [40], GSE36376 [41], GSE76427 [42], GSE25097 [43] and GSE63898 [44] datasets in the GEO database. We analyzed APEX1 and AFP mRNA levels in 46 sorafenib-resistant and 21 sorafenib-sensitive HCC patients from the GSE109211 dataset that were enrolled in the phase-3 STORM-trial to determine the safety and efficacy of sorafenib in HCC patients [45]. We also analyzed APEX1 and AFP mRNA levels in the HCC patients from the GSE125336 dataset who were resistant (n=3) or sensitive (n=3) to anti-PD-1 therapy [46]. Furthermore, we analyzed APEX1 expression using the UALCAN portal [47] in 371 primary HCC tissues from patients with different tumor grades and tumor stages and 50 adjacent normal liver tissues at the TCGA database.

**APEX1 protein expression analysis**

The APEX1 protein expression data from HCC and normal liver tissue samples at the TCGA database were obtained from The Human Protein Atlas (http://www.proteinatlas.org) database [48]. Immunohistochemical staining was performed using the anti-APEX1 antibody (Cat. No. sc-17774; Santa Cruz, USA).

### Real-time quantitative PCR (qPCR)

We obtained tumor and adjacent normal liver tissues from 45 HCC patients that underwent surgical resection at the Fuzhou General Hospital. All the patients gave informed and written consent. The study was approved by the institutional review board of the Fuzhou General Hospital (IRB00006161). The samples were immediately stored at -80°C. Total RNA was extracted from the tumor and adjacent normal liver samples using Trizol, and diluted in DEPC-treated water. The cDNA synthesis was performed using SuperScript Reverse Transcriptase and quantified using the Nanodrop (ND-2000) by analyzing the A_260_/A_280_ ratio. Equal amounts of cDNA from different samples were subjected to qPCR using the Hieff^TM^ qPCR SYBR Green Master Mix (High Rox Plus). The APEX1 mRNA levels were normalized to GAPDH mRNA levels and the relative APEX1 expression was determined using the 2^-ΔΔCT^ method. The specific qPCR primers used were : APEX1 (forward primer: 5′-TCTTGGAATGTG GATGGGCT-3′; reverse primer: 5′-ACTGTACCCTTC CTTGTCCG-3′); GAPDH (forward primer: 5′-AGGTCGGAGTCAACGGATTT-3′; reverse primer: 5′-ATC TCGCTCCTGGAAGATGG-3′). All primers were synthesized by Invitrogen, and the specificity of the primers was confirmed by performing the melting curve analysis as previously reported [49].

### Receiver operating characteristic (ROC) curve analysis

The ROC curve analysis was performed using the Wilson/Brown method and 95% confidence interval for the HCC and adjacent normal liver tissues or liver cirrhosis tissues. The area under the ROC curve (AUC) value was estimated to determine the diagnostic significance of APEX1 expression in HCC patients compared to the control group.

### Kaplan-Meier survival curve analysis

The 5-year overall survival (OS) rates were determined for HCC patients using Kaplan Meier database [50]. We divided 364 HCC patients into high- and low-APEX1 expression groups. The best cut-off value was determined from all possible cut-off values between the lower and the upper quartiles [50]. We also determined the false discovery rate and the corresponding *p*-value to ensure that the most optimal cutoff value was used. The follow-up time was set to 60 months following which the median survival time was determined for the HCC cohort. The patients that were still alive at the 60 month follow-up were censored.

### Gene set enrichment analysis (GSEA)

We performed GSEA analysis of HCC and normal liver tissue samples from the TCGA database to determine the enriched genes. Moreover, the HCC samples were subdivided into high and low APEX1 expression groups based on the median APEX1 expression value, which were also analyzed with GSEA. The functional gene set file “c5. all. v7.0. symbols. gmt” was used to determine the enriched genes in HCC tissues. The p value for all the pathways was determined after 1000 permutations. Pathway enrichment was determined in a weighted manner. The pathways and genes with a *P*-value <0.05 and FDR <0.25 were considered significantly enriched.

### Statistical analysis

The unpaired Student’s *t*-test was used to determine statistically significant differences in APEX1 expression between HCC tissues and adjacent normal liver or liver cirrhosis tissues. Paired Student’s *t*-test was used to analyze APEX1 expression data in HCC and adjacent normal liver tissues samples from the GSE76427 dataset and 45 fresh samples collected at our hospital. Kaplan-Meier survival curve analysis and log-rank test was used to determine the survival rate. Correlation analysis was performed by the Pearson method and the Pearson correlation coefficients were analyzed by the two-tailed t-test. ROC curves were analyzed by the Wilson/Brown method and 95% confidence intervals to determine differences between HCC and adjacent normal liver or liver cirrhosis tissues. The area under the ROC curve (AUC) value was used to determine the diagnostic significance of APEX1 and AFP expression in HCC tissues compared with the control group. All the results were analyzed using the GraphPad Prism version 8 software. A *p*-value below 0.05 was considered statistically significant.

### Ethics approval

The study was approved by the institutional review board of Fuzhou General Hospital (IRB00006161).

## Supplementary Material

Supplementary Figures

Supplementary Table 1
