# Supplementary material for: APEX1 is a novel diagnostic and prognostic biomarker for hepatocellular carcinoma
Source: Aging (Albany NY). 2020 Mar 13;12(5):4573–91. doi: 10.18632/aging.102913 (PMC7093175; doi:10.18632/aging.102913)
Supplement: Supplementary Figures [file aging-12-102913-s002..pdf]

SUPPLEMENTARY FIGURES

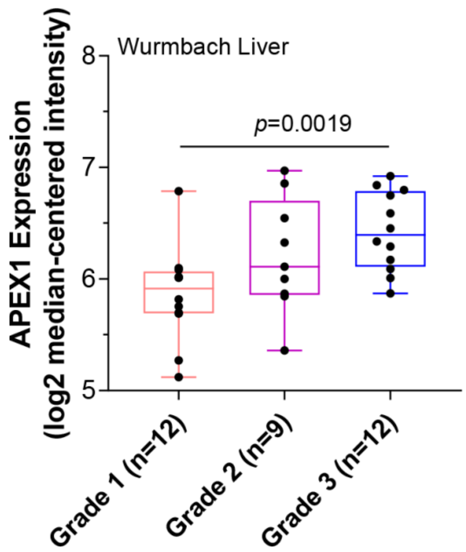

Supplementary Figure 1. The Wurmbach Liver with different tumor grade was subjected to analyze the levels of APEX1.

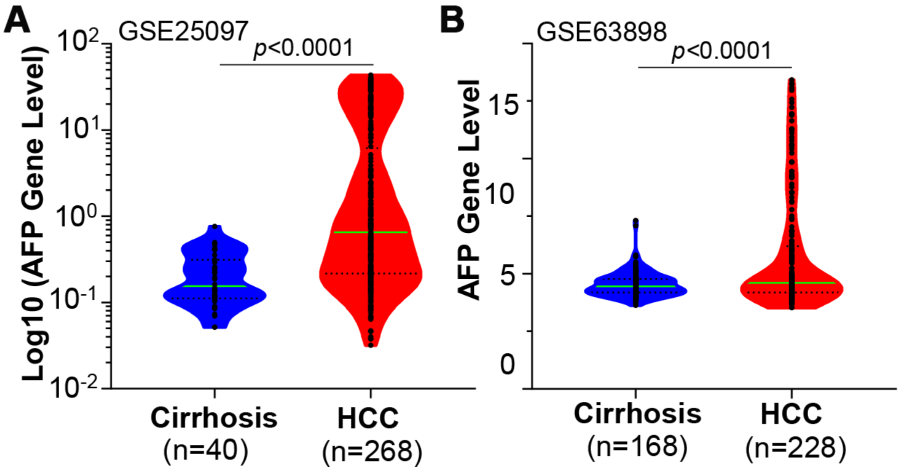

Supplementary Figure 2. (**A**) The levels of AFP were evaluated between cirrhosis and liver tumor in GSE25097 and (**B**) GSE63898.

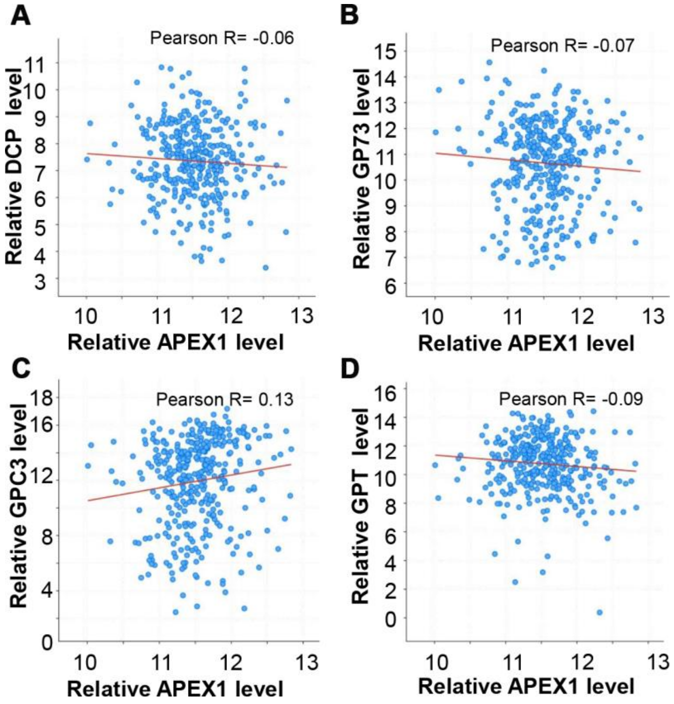

Supplementary Figure 3. The correlation analysis between APEX1 and (**A**) DCP, (**B**) GP73, (**C**) GPC3 and (**D**) GPT was conducted in cBioPortal for Cancer Genomics with TCGA liver cancer data.

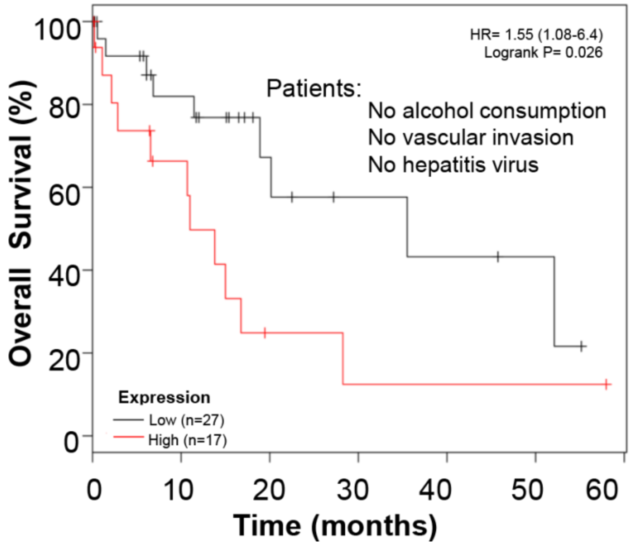

Supplementary Figure 4. The patients without alcohol consumption, tumor vascular invasion and hepatitis virus infection were conducted to evaluate the overall survival rate according to the expression of APEX1.
